# Highly selective plasma-activated copper catalysts for carbon dioxide reduction to ethylene

**DOI:** 10.1038/ncomms12123

**Published:** 2016-06-30

**Authors:** Hemma Mistry, Ana Sofia Varela, Cecile S. Bonifacio, Ioannis Zegkinoglou, Ilya Sinev, Yong-Wook Choi, Kim Kisslinger, Eric A. Stach, Judith C. Yang, Peter Strasser, Beatriz Roldan Cuenya

**Affiliations:** 1 Department of Physics, University of Central Florida, Orlando, Florida 32816, USA; 2 Department of Physics, Ruhr-University Bochum, 44780 Bochum, Germany; 3 Department of Chemistry, Chemical Engineering Division, Technical University Berlin, 10623 Berlin, Germany; 4 Chemical and Petroleum Engineering and Physics, University of Pittsburgh, Pittsburgh, Pennsylvania 15261, USA; 5 Center for Functional Nanomaterials, Brookhaven National Laboratory, Upton, New York 11973, USA

## Abstract

There is an urgent need to develop technologies that use renewable energy to convert waste products such as carbon dioxide into hydrocarbon fuels. Carbon dioxide can be electrochemically reduced to hydrocarbons over copper catalysts, although higher efficiency is required. We have developed oxidized copper catalysts displaying lower overpotentials for carbon dioxide electroreduction and record selectivity towards ethylene (60%) through facile and tunable plasma treatments. Herein we provide insight into the improved performance of these catalysts by combining electrochemical measurements with microscopic and spectroscopic characterization techniques. Operando X-ray absorption spectroscopy and cross-sectional scanning transmission electron microscopy show that copper oxides are surprisingly resistant to reduction and copper^+^ species remain on the surface during the reaction. Our results demonstrate that the roughness of oxide-derived copper catalysts plays only a partial role in determining the catalytic performance, while the presence of copper^+^ is key for lowering the onset potential and enhancing ethylene selectivity.

## Abstract (web summary)

Carbon dioxide electroreduction is a promising route to hydrocarbon synthesis, but more efficient and selective catalysts are needed. Here the authors show that plasma-activated copper can catalyse the reduction of carbon dioxide to ethylene with high efficiency and reveal cationic copper as the active site.

## Graphical abstract

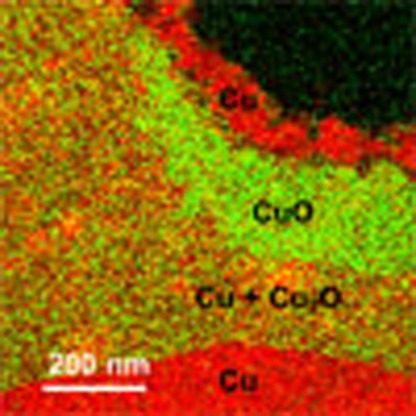

The development of technologies to capture and convert carbon dioxide (CO_2_)—a greenhouse gas released by the burning of fossil fuels—to useful products is critically needed to mitigate global warming. The electrocatalytic reduction of CO_2_ (CO_2_RR) shows promise as a viable CO_2_ utilization process since it can occur under ambient conditions and low cost copper (Cu) catalysts can facilitate the reduction pathway to hydrocarbons^1^,^2^,^3^. However, more efficient catalysts are still required because large overpotentials are required for bulk Cu to reduce CO_2_ to hydrocarbon products (methane and ethylene) while competing with the hydrogen evolution side reaction^4^,^5^,^6^,^7^,^8^. Efficient Cu catalysts with high selectivity to ethylene are especially desirable due to the value of ethylene as a widely used chemical feedstock.

Investigations in recent years have led to the development of many interesting metal-based catalytic materials for CO_2_RR. Nanostructured catalysts such as nanoparticles^9^,^10^,^11^,^12^,^13^,^14^, nanocubes^15^,^16^, nanofoams^17^ and nanowires^18^ have shown vastly improved activity or selectivity over bulk materials^19^,^20^. Recently, nanostructures derived from the reduction of copper oxides have shown vastly improved CO_2_ reduction efficiency at lower overpotentials^21^,^22^,^23^,^24^. These materials were synthesized from the reduction of thermally oxidized Cu or electrodeposited copper(I) oxide (Cu_2_O), and in general display improved current density, enhanced CO_2_ reduction to carbon monoxide (CO) at low overpotentials, and a partial suppression of methane (CH_4_) in favour of ethylene (C_2_H_4_) at higher overpotentials. Despite these promising results, the mechanism behind the reactivity trends of oxide-derived CO_2_RR catalysts is still under dispute. Grain boundaries in oxide-derived Cu have been suggested to support unique surface sites which may be active for CO_2_RR^22^,^25^,^26^. Another mechanism could be the increase in local pH due to high current densities^27^ on the highly roughened surfaces, which could alter the reaction pathway in favour of ethylene^28^,^29^,^30^. Alternatively, the nanostructuring of the catalyst surface during the oxide reduction may also provide low-coordinated atoms as active sites^9^. Cu^δ+^ sites which may remain on the catalyst surface during the reaction have also been suggested to be the active sites^31^,^32^,^33^,^34^,^35^. However, no operando evidence of this claim exists up to this point, and only *ex situ* evidence has been presented. This *ex situ* evidence is complicated by the rapid formation of native oxides on metallic Cu surfaces upon removal from the reactor and exposure to air^23^. In general, it has been assumed that Cu oxides are completely reduced during the relatively high CO_2_RR potentials used (that is, ∼1 V versus reversible hydrogen electrode (RHE)), and that the reaction occurs only on metallic Cu species.

Oxygen plasma treatment is a facile and scalable technique to controllably oxidize and nanostructure metal catalysts for CO_2_RR that has not yet been explored. Plasma treatment is a powerful method to activate catalysts, for example by removing capping ligands used in the synthesis of nanoparticles without sintering^36^,^37^, by rapidly changing the chemical state of the surface at room temperature^38^,^39^, or by creating defects or embedded atoms which can improve reactivity^40^. We have used oxygen (O_2_) and hydrogen (H_2_) plasmas to create novel nanostructured oxide layers and porous surfaces with tunable morphology and chemical state on polycrystalline Cu. These catalysts can lower the onset potential of CO_2_ reduction to ethylene by 350 mV compared with electropolished Cu, with a maximum faradaic selectivity of >60% at −0.9 V versus RHE. Here, using a combination of characterization techniques—including operando X-ray absorption fine-structure spectroscopy (XAFS), scanning transmission electron microscopy (STEM) equipped with energy dispersive X-ray spectroscopy (EDS)—we gain insight into the catalysts in their working state and find that Cu^+^ is the active species for reducing CO_2_ to ethylene. Through our results, we unravel the mechanism behind the improved activity and unique ethylene selectivity of oxide-derived Cu catalysts and lay out the design principles necessary for improved ethylene-selective CO_2_RR catalysts.

## Results

### Synthesis and characterization

**Figure 1:**
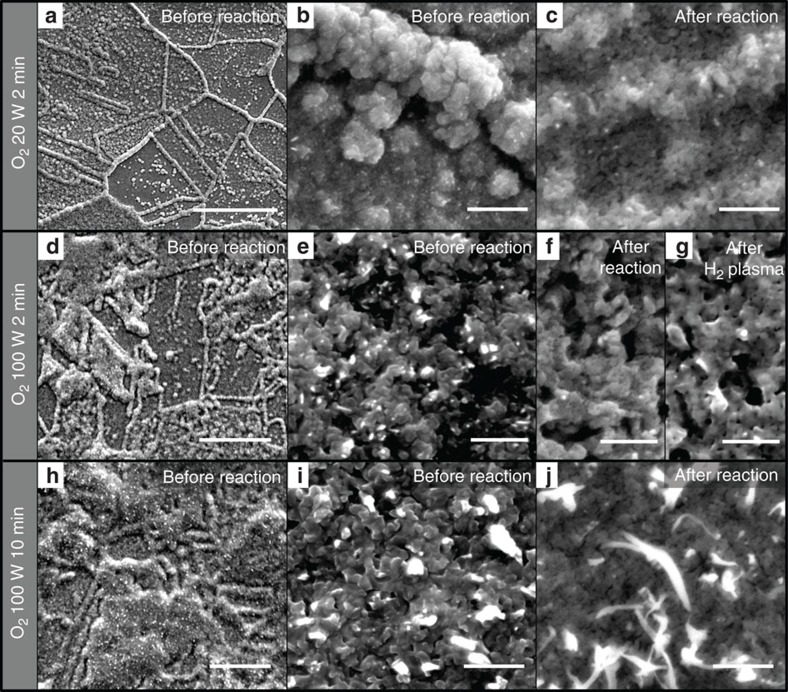
Morphological characterization of plasma-treated Cu foil electrodes. SEM images of Cu foils treated with O_2_ plasma for (**a**–**c**) 20 W 2 min; (**d**–**g**) 100 W 2 min; and (**h**–**j**) 100 W 10 min. (**a**,**b**,**d**,**e**,**h**,**i**) The morphology of the as-prepared foils. (**c**,**f**,**j**) The morphology after the CO_2_RR reaction. (**g**) The sample plasma treated with O_2_ at 100 W for 2 min after an additional H_2_ plasma treatment at 100 W for 10 min. Scale bars, (**a**) 10 μm; (**b**,**c**,**e**–**g**,**i**,**j**) 500 nm; (**d**,**h**) 20 μm.

To synthesize plasma-activated Cu, electropolished polycrystalline Cu foils were treated in O_2_ and H_2_ plasmas of varying power and duration. Figure 1 presents scanning electron microscopy (SEM) images of the Cu films treated with O_2_ plasma at 20 W for 2 min (top row) and 100 W for 2 min (middle row) and 10 min (bottom row). By tuning the plasma conditions, the morphology of the surface and oxide thickness can be easily controlled. Growth of the Cu oxide begins at grain boundaries, forming micron-sized wires as shown in Fig. 1a. With increasing plasma time and power, the wires merged, forming a highly roughened surface, (c.f. Fig. 1b,e,i) and became highly porous with an observed 64% increase in the number of pores and 50% decrease in pore size (Supplementary Figs 1 and 2). Subsequently, the plasma-activated Cu samples were partially reduced *in situ* during CO_2_RR, and the resulting surface morphology is shown in the last column of Fig. 1. For most samples, an increase in the porosity was observed. Interestingly, the sample oxidized at 100 W for 10 min showed the growth of hair-like nanoneedles after the reaction. To compare the effect of plasma reduction on the film morphology to the electrochemical reduction mentioned above, a H_2_ plasma was used (100 W for 10 min), and is shown in Fig. 1g. The resulting structure was very similar to the electrochemically reduced sample (c.f. Fig. 1f), indicating that plasma reduction or electrochemical reduction of the Cu oxides results in a similar structure. SEM images for additional plasma treatments are shown in Supplementary Fig. 1.

**Figure 2:**
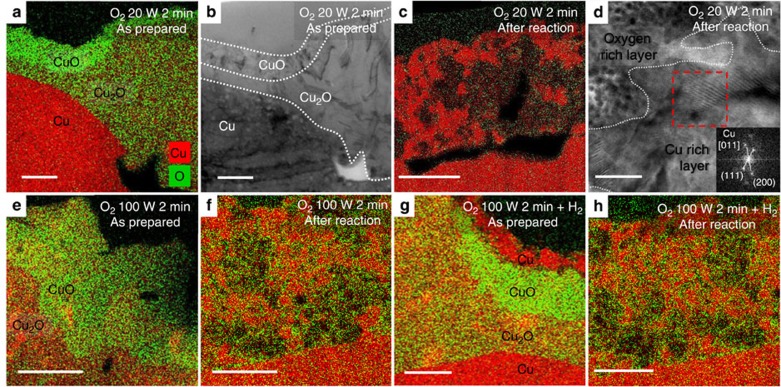
Morphological and chemical analysis of plasma-activated Cu foils. EDS elemental maps of Cu foils treated with O_2_ plasma for (**a**–**d**) 20 W 2 min; (**e**–**f**) 100 W 2 min; and (**g**–**h**) 100 W 2 min+H_2_ plasma. The images labelled ‘after reaction' were used as catalyst for CO_2_RR for 1 h at −0.91 V versus RHE. (**b**,**d**) HRTEM and SAED analysis of the O_2_ plasma 20 W 2 min treated sample before and after the reaction, respectively. Scale bars, (**a**–**c**) 300 nm; (**d**) 20 nm; (**e**–**h**) 200 nm.

To gain further information on the structure and chemical state of the plasma-treated Cu films, cross-sections of the samples were measured using STEM combined with elemental mapping using EDS. Cross-sectional EDS maps of plasma-treated Cu samples before and after CO_2_RR are shown in Fig. 2 and Supplementary Fig. 3. After the O_2_ plasma treatment, the EDS maps show two well-defined oxide layers over the bulk metallic Cu film. Stoichiometric analysis using EDS data (Supplementary Table 1) resulted in Cu:O ratios of 2.7:1.0 and 1.3:1.0 for the interlayer and upper layers, respectively, indicating that the interlayer is Cu_2_O, while the upper layer is copper(II) oxide (CuO). It is also apparent that at grain boundaries and crystallographic domains with the fastest oxide growth, a thick 1.5-μm Cu_2_O interlayer grows with a thinner 100 nm CuO top layer, while in domains with slower oxide growth, the CuO layer is thicker at 260 nm—as clearly shown in Fig. 2a. Figure 2g shows the 100 W 2 min oxidized Cu with a subsequent H_2_ plasma treatment. A 100 nm layer at the surface is reduced entirely to metallic Cu, with the CuO and Cu_2_O oxide layers being still observed subsurface. After H_2_ plasma treatment, the sample was exposed to air for more than a week before TEM sample preparation and analysis, and the native Cu oxide layer, measured to be <3 nm thick, was insignificant compared with the thick oxide layers which formed from the plasma treatment and remained during the reaction. This indicates that the observed oxides in our samples before or after reaction are not simply due to surface oxidation in air during the *ex situ* sample transfer to the TEM. Figure 2c,f,h also shows EDS maps of these samples after 1 h of CO_2_RR at −0.91 V versus RHE. Remarkably, after the reaction a single layer with O atom concentration ranging from 3 to 29 atomic % remains with porous and oxygen-rich regions observed. The Cu oxide species are present throughout the layer, indicating that the surface is rich in Cu^+^ sites which remain stable on the surface and near surface layers during the reaction. This finding challenges the conventional assumption that only metallic Cu (Cu^0^) is the active species during CO_2_RR and indicates that Cu^+^ plays a significant role in the unique reactivity of oxide-derived Cu.

Figure 2b,d and Supplementary Fig. 4 show high-resolution TEM (HRTEM) and selected area electron diffraction (SAED) patterns of the O_2_ 20 W 2 min plasma-treated sample before and after the reaction. The diffraction analysis confirms that a CuO top layer and Cu_2_O interlayer exist before the reaction (Fig. 2b). After the reaction, the layer transforms into a mixture of Cu-rich regions, which contain only 3.4±0.9% oxygen atoms, and oxygen-rich regions (Supplementary Fig. 4) with 19.5±2.6% oxygen atoms identified as Cu_2_O. It is clear that the oxygen is depleted during the reaction, but the surface layer of the oxidized Cu films is still rich in oxygen even after 1 h of reaction. It is unlikely that the Cu oxides in the sample after reaction could be a result of air exposure, since the native oxide would be much thinner due to its slow growth rate at room temperature in air and would contain Cu(II) species, which are not observed here.

### Operando X-ray absorption spectroscopy

**Figure 3:**
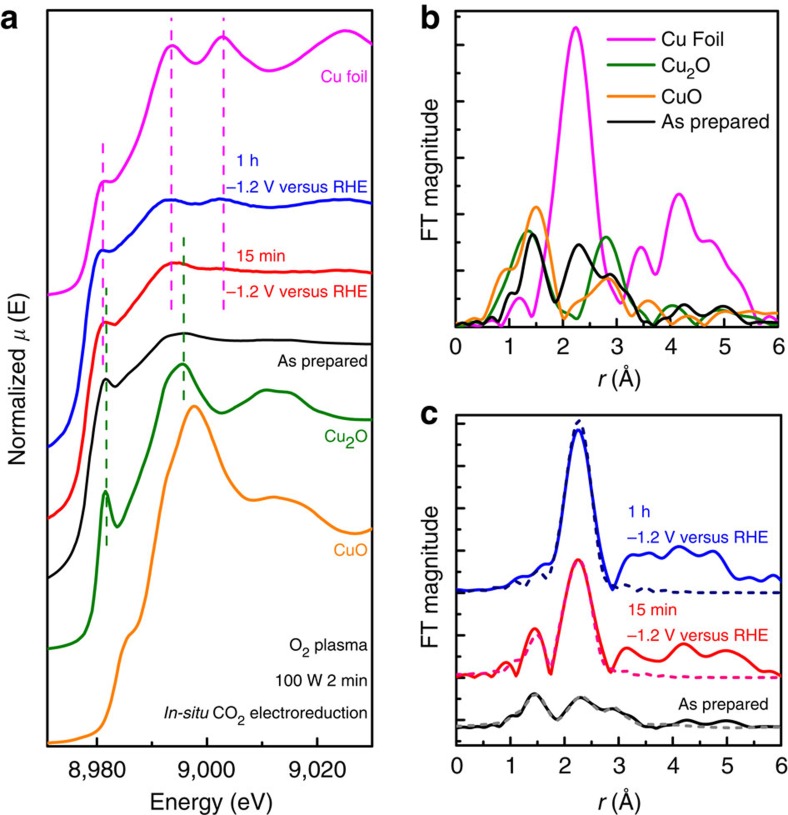
Operando structural and chemical characterization during CO_2_ electroreduction. (**a**) XANES spectra of the O_2_ 100 W 2 min treated sample measured under operando conditions in 0.1 M KHCO_3_ during the first 15 min and after 1 h of reaction at −1.2 V versus RHE. Bulk Cu, Cu_2_O and CuO spectra are plotted as reference. (**b**) EXAFS spectrum of the as-prepared sample plotted with references. (**c**) EXAFS spectra and fits (dashed lines) of the sample measured under operando conditions. Fourier transforms are *k*^2^-weighted. Fit results are given in Supplementary Table 2.

To further investigate the changes in the structure of the oxide layer during CO_2_RR and gain insight into the chemical state of the active Cu species, operando X-ray absorption near-edge structure (XANES) and extended XAFS (EXAFS) were used. Figure 3 shows the Cu K-edge XANES and EXAFS spectra of the O_2_ 100 W 2 min plasma-treated Cu measured in fluorescence at small incidence angle (for enhanced surface sensitivity) during CO_2_RR at −1.2 V versus RHE. Although the thickness of the sample caused dampening of the XAFS signals due to self-absorption, the XANES spectrum of the as-prepared plasma-oxidized Cu shows the features of Cu_2_O, in particular, the prominent shoulder at the edge marked with a green dashed line at ∼8,982 eV. The presence of metallic Cu and Cu oxide in the initial sample is also clear in the EXAFS data shown in Fig. 3b,c. During the first 15 min of reaction, a combination of Cu and Cu_2_O features are still present, and at 1 h, only metallic Cu features are discernable. Although an oxygen content of ∼25–28% is still present in the surface layer of the catalyst after 1 h of reaction according to STEM-EDS, XAFS probes further into the bulk, and when the films are partially reduced, the XAFS signals become dominated by the signal of the metallic Cu underlayer. Nevertheless, we have shown here unquestionably that for our plasma-oxidized Cu catalyst, Cu_2_O species are present and can participate in the reaction. It is clear that significant changes occur in the bulk of the Cu oxide layer during the first 15 min of reaction in which the initial CuO and Cu_2_O layers are reconstructed and some of the oxygen is depleted. After this initial change, it is likely that the Cu_2_O species remain relatively stable in the surface layer, since the activity and selectivity measured after 10 min and 1 h are unchanged for all the oxidized samples, see Supplementary Fig. 5. Furthermore, the reactivity of the O_2_ 20 W 2 min sample was observed to be stable over the course of 5 h, suggesting there is no significant change on the active phase during this time (Supplementary Fig. 6). Therefore, it is likely that Cu^+^ species can be supplied to the surface from the thick initial oxide surface and subsurface layers during the reaction to maintain the reactivity of these catalysts over time.

### CO_2_ electroreduction performance

**Figure 4:**
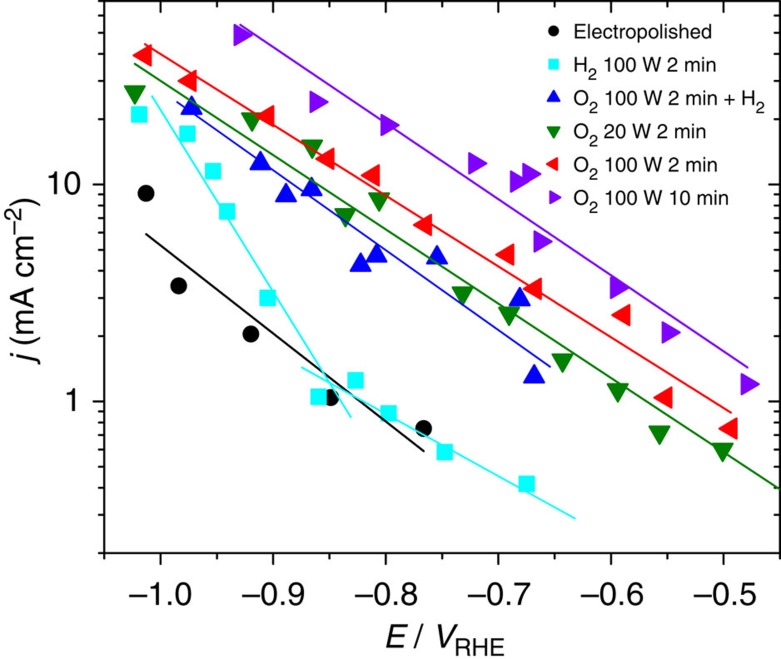
Electrochemical activity during CO_2_ electroreduction. Geometric reduction current density as a function of applied potential.

The catalytic activity and selectivity of the plasma-activated copper foils were studied by performing bulk electrolysis in 0.1 M potassium bicarbonate (KHCO_3_) at a constant potential and analysing the reaction products via chromatographic techniques. Figure 4 shows the geometric current density as a function of applied potential. The plasma treatment has a clear effect on the activity of Cu towards CO_2_RR which can partially be attributed to the surface roughness. In general, longer and higher power O_2_ plasma treatment increases the roughness factor (Table 1), which is an estimate of the active surface area derived by measuring the double layer capacity and normalizing by the electropolished foil (Supplementary Table 3; Supplementary Fig. 7). However, roughness effects alone cannot explain all of our experimental findings satisfactorily. In fact, the initial oxidation state of the sample also affects the current density. This is particularly evident when we compare the catalytic activity of the O_2_ 100 W 2 min and O_2_ 100 W 2 min+H_2_ plasma-treated Cu. Although both samples have a similar surface roughness, as shown in Table 1, their catalytic activity is significantly different. The H_2_ plasma treatment on the oxidized sample reduces the activity by about two times compared with the oxidized sample without H_2_ treatment. The H_2_ plasma treatment depletes the oxide species available initially in the sample, as shown in Fig. 2g, while having minimal effect on the surface roughness. This result is an indication that the Cu oxide species are critical for improved current density. As demonstrated by our STEM-EDS data, even after 1 h of CO_2_RR, patches of Cu_2_O are still observed near the sample surface.

**Figure 5:**
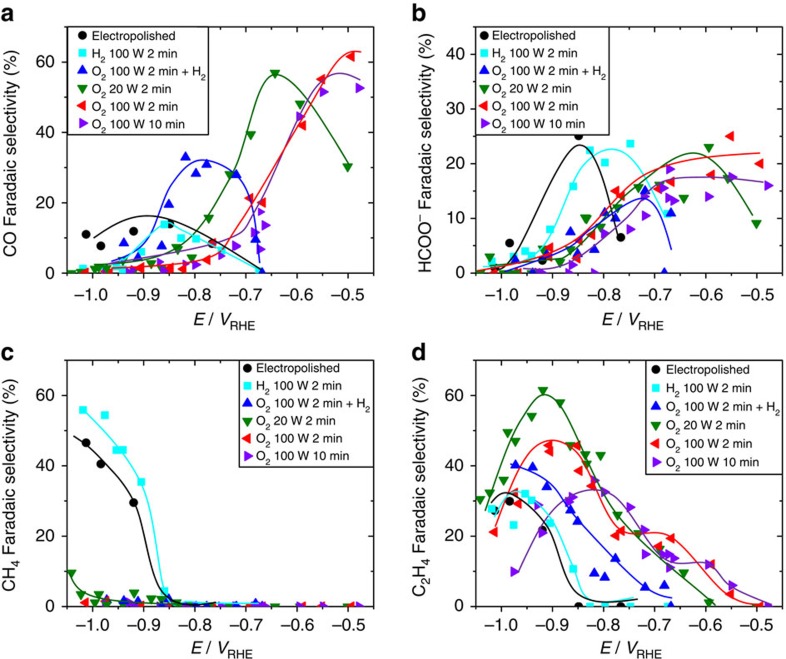
Faradaic selectivities of the main CO_2_RR products. Data were acquired after 60 min of CO_2_ electrolysis at a constant potential in CO_2_ saturated 0.1 M KHCO_3_. (**a**) CO, (**b**) formate, (**c**) CH_4_ (**d**) C_2_H_4_. Solid lines are guides for the eye. The remaining selectivity is due to H_2_, which is shown in Supplementary Fig. 8.

Figure 5 shows the faradaic selectivity of the CO_2_RR products for the plasma-activated Cu samples as a function of applied potential. The plasma treatments not only affect the activity of copper, but more importantly, they also have a remarkable effect on selectivity. The selectivity change is most drastic on the O_2_ treated samples: on one hand, this treatment clearly suppresses methane formation (Fig. 5c), while on the other hand, it enhances the formation of the other products of CO_2_RR (CO, formate and ethylene). Consistent with results from Kanan and co-workers^22^, we observe that the onset potential towards CO and formate (HCOO^−^) is shifted to lower overpotentials. Furthermore, for our plasma-oxidized Cu, the selectivity towards CO reaches a maximum of 60% at −0.5 V versus RHE, which is three times higher than for electropolished Cu foil. In our study, the onset of ethylene production is also shifted from approximately −0.85 V versus RHE for metallic Cu foils to as low as −0.5 V versus RHE for the most oxidized Cu foils. In addition, we observe a remarkably high selectivity towards ethylene, reaching 60% at −0.9 V versus RHE for the O_2_ 20 W 2 min plasma-treated sample. This value is higher than other oxide-derived Cu catalysts synthesized from thermal oxidation^22^, which show <10% ethylene efficiency at these potentials, or from electrodepositon of Cu_2_O^21^,^23^,^24^. In addition, the most oxidized foils in this study also produce trace amounts of ethanol (Supplementary Fig. 8). Interestingly, the 100 W O_2_ plasma-treated samples, which have been exposed to a stronger oxygen plasma treatment and are characterized by the highest roughness factors, also produce trace amounts of ethane, but nonetheless exhibit lower C_2_H_4_ selectivities. These results indicate that there is an optimal oxidation treatment to favour ethylene formation, in agreement with results from Ren *et al*^21^. We hypothesize that this trend could be related to mass diffusion limiting ethylene formation on the most roughened surfaces where high geometric currents are achieved. This is also consistent with our observation that at high overpotentials—at which high geometric current densities are also achieved—ethylene selectivity is suppressed.

To confirm that the selectivity trend for the oxidized samples is due to the oxidation state, not the surface roughness, we can compare the O_2_ 100 W 2 min treated sample with the same sample which was exposed to an identical O_2_ plasma treatment plus H_2_ plasma. Despite their similar roughness (Table 1), their selectivity is drastically different. On both samples, methane formation is suppressed, however on the former sample, which was only treated with O_2_ plasma, the onset potential for the formation of CO, HCOO^−^ and C_2_H_4_ is clearly shifted to less negative potentials. The main difference between these two samples is the availability of Cu^+^ sites in their initial state. Thus, by depleting the Cu^+^ sites using the H_2_ plasma, we have reduced the number of active sites available for C_2_H_4_ formation, and therefore altered the selectivity.

We also observed that the H_2_ 100 W 2 min plasma-treated sample, which has a similar roughness to the electropolished Cu foil, has an improved catalytic performance towards CO_2_RR compared with the polished foil. The H_2_ plasma-treated sample exhibits an earlier onset potential for formate production and a higher faradaic selectivity towards methane. An explanation for this effect could be improved CO_2_ reduction on defect sites generated by the plasma treatment, as shown in Supplementary Fig. 1. Another possible mechanism could be cleaning of contaminants or the native oxide from the Cu surface by the H_2_ plasma, which may improve the CO_2_RR performance in comparison to the electropolished sample. XPS shows that the small adventitious carbon 1s peak present on the electropolished foil is diminished after the H_2_ plasma treatment, although the oxidation state of the surface of the foil is not changed (Supplementary Fig. 9).

## Discussion

**Figure 6:**
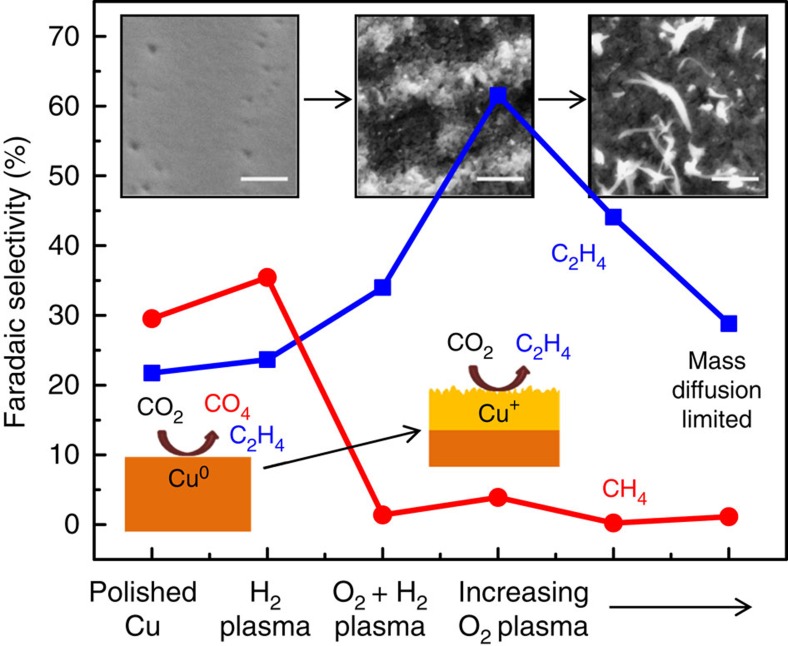
Summary of hydrocarbon selectivity of plasma-treated Cu foils. From left to right, the insets show SEM images of the low surface area H_2_ plasma-treated metallic Cu foil, the O_2_ 20 W 2 min plasma-treated Cu foil with optimal ethylene selectivity, and the high surface area nanoneedles on the O_2_ 100 W 10 min oxidized sample after the reaction (500 nm scale bars).

Figure 6 shows a summary of the hydrocarbon selectivity as a function of the plasma treatment of the Cu foils. Two competing effects are controlling the reactivity of the plasma-treated Cu foils: the surface roughness and the oxidation state of the surface layer. As described above, after oxidizing the surface layer with O_2_ plasma, we see an almost complete suppression of methane formation. As the foils are more oxidized, the surface roughness increases, and there is a drop in the ethylene selectivity, likely due to mass diffusion limitations.

Previous studies have proposed that the catalytic behaviour of such oxygen-derived Cu catalysts can be attributed to a roughness effect or to the presence of strongly binding defect sites such as grain boundaries. Recent work in Verdaguer-Casadevall *et al*.^25^ have shown that CO-binding energy on oxide-derived Cu is higher than on metallic copper. This high-binding energy could explain the early onset potential for CO_2_RR, but does not explain why subsequent H_2_ plasma treatment would suppress the ethylene selectivity of the 100 W 2 min oxidized sample in our study.

Another proposed mechanism for the behaviour of oxide-derived CO_2_RR catalysts is a pH effect. It is expected that during the catalytic reaction on these rough surfaces there is a significant rise in the local pH, suppressing the pH-dependent CO protonation that leads to methane formation^41^,^42^. The pH-independent pathway via CO dimerization, however, is not affected, resulting in high ethylene selectivities^28^,^43^,^44^. Consistently, all samples with some initial oxidation treatment (rough surfaces) exhibit suppression of methane, as shown in Fig. 6, which may be attributed to the pH effect. However, the lower onset potential for ethylene production observed in the oxygen-treated samples (Fig. 5d) cannot be attributed to a local pH effect. In fact we observed that that the increasing oxidation treatment leads to a decrease in the overpotential for C_2_H_4_ production, indicating that high activity for ethylene can be related to the oxidation–reduction treatment and not to a local pH effect.

By comparing the catalytic performance of O_2_ plasma 100 W 2 min+H_2_ and O_2_ plasma 100 W 2 min Cu, we have demonstrated that purely a roughness effect cannot explain the reactivity trends of our plasma-activated oxide-derived Cu catalysts, since these catalysts with nearly the same roughness, but different oxide content have significantly different reactivity and selectivity. In addition, while the high local pH could explain the suppression of CH_4_, it cannot explain the earlier onset potential for CO_2_RR. Since surface roughness and pH effects cannot fully explain the reactivity of our plasma-activated Cu, it is clear that another mechanism is also involved in controlling the reactivity. Based on our STEM-EDS and operando XAFS measurement we conclude that, contrary to common belief, Cu^+^ species are stable under reaction conditions and play a determining role in the reactivity of oxide-derived Cu. These species may interact with negatively charged CO_2_ reduction intermediates which may play an important role in determining selectivity.^41^

The mechanism behind the stability of Cu^+^ species under the thermodynamically unfavourable conditions of CO_2_RR is still under investigation, however, several mechanisms may explain this phenomenon. One possibility is that the highly roughened surfaces with low-coordinated sites which are formed after O_2_ plasma treatment may bind more strongly to oxygen, helping to stabilize oxides during the reaction. Strain in the surface caused by the oxidation and reduction of the surface may also cause strong oxygen binding. The highly porous nature of the films formed might also serve as favourable oxygen reservoir. Another possibility is that the high local pH could help to stabilize Cu^+^ by negatively shifting the overpotential for Cu_2_O reduction. Recent studies of nanostructured tin oxide catalysts for CO_2_ electroreduction have also suggested that oxide species are stable during the reaction and play a controlling role in their selectivity. Sn oxides and not metallic Sn were shown to be the active species for CO_2_ reduction to formic acid and are metastable during the reaction at reducing potentials, even though the oxides are thermodynamically unstable under these conditions^45^,^46^,^47^. While similar stability of Cu^+^ has been suggested for oxide-derived Cu catalysts^23^,^31^,^48^, we provide here the first direct evidence that Cu^+^ species are stable during CO_2_RR and are key for controlling selectivity.

In summary, we have synthesized superior oxygen-derived Cu catalysts using plasma treatments and used a synergistic combination of advanced *ex situ*, *in situ* and operando techniques to reveal the key mechanisms behind the improved performance of oxide-derived copper catalysts. Plasma treatment was found to be a facile method to rapidly oxidize Cu foils, which resulted in stable oxide layers with a unique surface structure. These plasma-oxidized catalysts achieve lower onset potentials for CO, formate and ethylene, as well as outstanding ethylene selectivity of 60% at −0.9 V versus RHE. In addition, using STEM-EDS and operando XAFS, we have found that the oxides in the surface layer are surprisingly stable against reduction, with a significant amount of oxide species and dissolved oxygen remaining after 1 h of reaction at relatively high potentials (−0.91 V versus RHE). Furthermore, ethylene synthesis and methane suppression are due to the presence of these oxides. It is expected that the fundamental understanding extracted from our experimental study could aid the further design and optimization of oxide-derived Cu catalysts.

## Methods

### Catalyst synthesis

All Cu foils were initially electropolished in 85% phosphoric acid at 3 V versus a titanium foil, then thoroughly rinsed with ultra-pure water and dried with nitrogen. Plasma treatments were performed in 400 mtorr of H_2_ or O_2_ for the indicated plasma power and time. For the O_2_ 100 W 2 min+H_2_-treated sample, 100 W 10 min of H_2_ plasma was used.

### Electron microscopy

SEM images were obtained using a Quanta 200 FEG microscope from FEI with a field emitter as electron source. A secondary electron (Everhart-Thornley) detector was used for the image acquisition. An electron acceleration voltage of 10 kV and a working distance of 10 mm were chosen for the measurements. TEM cross-section samples were prepared using a FEI Helios 600 Dual Beam Focused Ion Beam (FIB) at the Center for Functional Nanomaterials (CFN) at Brookhaven National Laboratory (BNL). The FIB sectioning was performed at 30 keV with final milling at 5 KeV. Spatially resolved elemental maps were acquired using a FEI Titan TEM/STEM microscope with ChemiSTEM technology (X-FEG and SuperX with four windowless silicon drift EDS detectors) operated at 200 kV, at the National Center for Electron Microscopy (NCEM), Molecular Foundry at Lawrence Berkeley National Laboratory (LBL). A Hitachi 9500 TEM at the Nanoscale Fabrication and Characterization Facility (NFCF), University of Pittsburgh operated at 200 keV was used to acquire HRTEM images and SAED patterns.

### X-ray absorption spectroscopy

XANES and EXAFS spectra were measured at beamline 2–2 of the Stanford Synchrotron Light Source. A home-built operando electrochemical cell was used, with a platinum foil counter electrode and silver/silver chloride (Ag/AgCl) reference electrode. The samples were mounted behind an X-ray kapton window with 1 mm of electrolyte between the sample and window. The electrolyte (0.1 M KHCO_3_) was circulated between the cell and a reservoir in which CO_2_ was continuously bubbled. Measurements were performed in fluorescence at small incidence angle using a passivated implanted planar silicon (PIPS) detector. The acquisition of each spectrum took 15 min, and they were acquired during the first 15 min and after 1 h of reaction.

Data analysis was performed using the Athena and Artemis software. The FEFF8 code was used to simulate Cu, Cu_2_O and CuO spectra for EXAFS fitting. Further details are given in the Supplementary Table 2.

### Electrochemical measurements

Electrochemical measurements were carried out in a custom made two compartment cell, separated by a Nafion membrane. The glassware was cleaned in a ‘nochromix' bath and afterwards in concentrated HNO_3_ for 1 h, respectively, rinsed and sonicated with ultra-pure water several times. The working compartment was filled with 120 ml 0.1 M KHCO_3_ (Sigma-Aldrich ⩾99.95%). Before and during the electrochemical reaction the cell was purged continuously with CO_2_ (30 ml min^−1^), reaching a stable pH value of 6.8.

A platinum mesh 100 (Sigma-Aldrich 99.9%) was used as counter electrode and a leak-free Ag/AgCl electrode as reference electrode (Hugo Sachs Elektronik Harvard apparatus GmbH). The plasma-treated Cu foils were used as working electrode and contacted by a gold clamp. Every measurement was started with a linear voltammetric sweep, performed with a scan rate of −5 mV s^−1^ between *E*=+0.05 V/RHE and the working potential (between −0.45 V and −1.0 V/RHE) followed by a chronoamperometric step for 60 min. All reported potentials are corrected for Ohmic drop determined by electrochemical impedance spectroscopy.

### Product analysis

After 10 and 60 min of bulk electrolysis at constant potential, a sample of the gas was analysed by gas chromatography (Shimadzu GC 2016) to determine the production rate and faradaic selectivity of the gaseous products. In addition, an aliquot of the electrolyte was analysed by a high-performance liquid chromatograph (Agilent 1200 series).

### Data availability

The data supporting the findings of this study are available within the article and its Supplementary information files. All other relevant source data are available from the corresponding author upon request.

## Additional information

**How to cite this article**: Mistry, H. *et al*. Highly selective plasma-activated copper catalysts for carbon dioxide reduction to ethylene. *Nat. Commun.* 7:12123 doi: 10.1038/ncomms12123 (2016).

## Supplementary Material

Supplementary Information Supplementary Figures 1-9, Supplementary Tables 1-3 and Supplementary References.

Peer Review File

## Figures and Tables

**Table 1:** Roughness factors estimated through capacitance measurements.

**Sample**	**Roughness factor**
Electropolished	1.0
H_2_ 100 W 2 min	1.5
O_2_ 20 W 2 min	26.4
O_2_ 100 W 2 min	43.7
O_2_ 100 W 2 min+H_2_	48.3
O_2_ 100 W 10 min	89.7
