# Corrigendum: Highly selective plasma-activated copper catalysts for carbon dioxide reduction to ethylene

**DOI:** 10.1038/ncomms12945

**Published:** 2016-09-12

**Authors:** Hemma Mistry, Ana Sofia Varela, Cecile S. Bonifacio, Ioannis Zegkinoglou, Ilya Sinev, Yong-Wook Choi, Kim Kisslinger, Eric A. Stach, Judith C. Yang, Peter Strasser, Beatriz Roldan Cuenya

Nature Communications 7 Article number: 1212310.1038/ncomms12123 (2016); Published 06 30 2016; Updated 09 12 2016

An incorrect version of the Supplementary Information was inadvertently published with this Article in which the image for Supplementary Fig. 7 was missing. The Article has now been updated to include the correct version of the Supplementary Information.

## 

