# Supplementary material for: Highly selective plasma-activated copper catalysts for carbon dioxide reduction to ethylene
Source: Nat Commun. 2016 Jun 30;7:12123. doi: 10.1038/ncomms12123 (PMC4931497; doi:10.1038/ncomms12123)
Supplement: Supplementary Information — Supplementary Figures 1-9, Supplementary Tables 1-3 and Supplementary References. [file ncomms12123-s1.pdf]

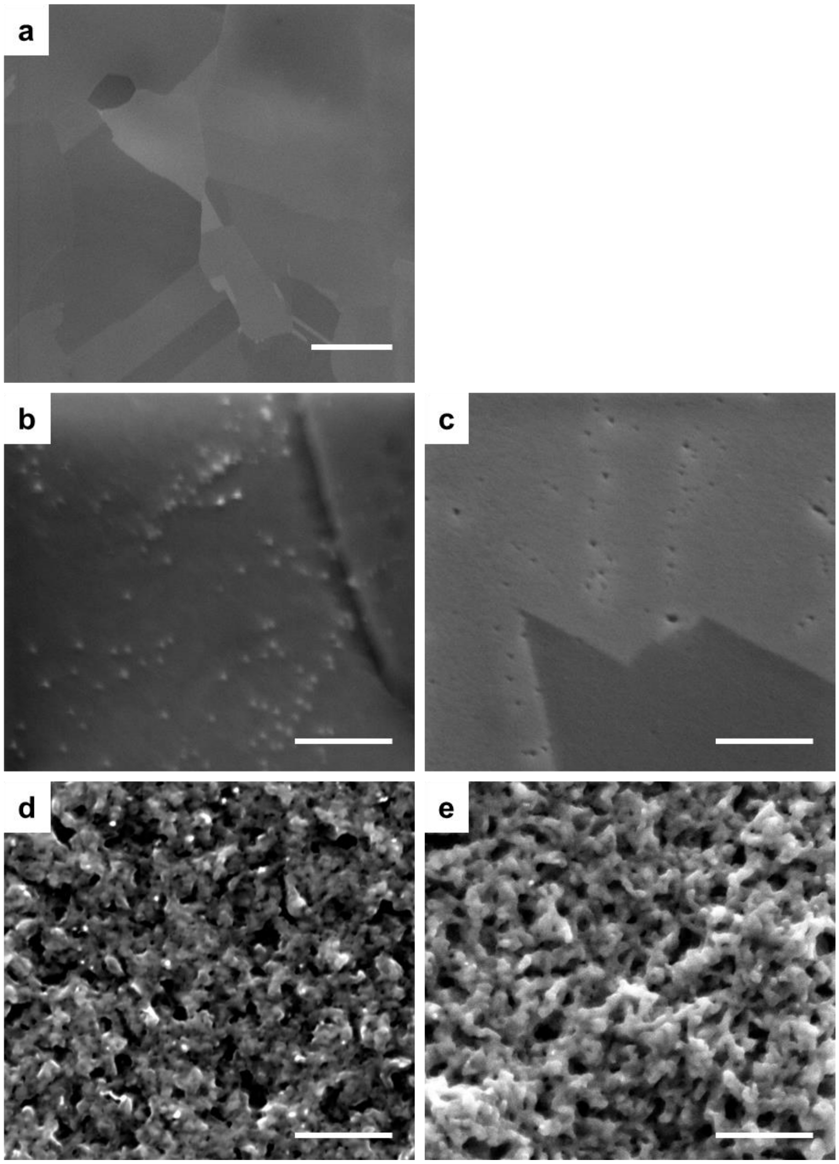

Supplementary Figure 1. a) SEM image of Cu foil after electropolishing (5 µm scale bar). SEM images of Cu foils treated with H_2 plasma at 100W for 2 minutes b) as prepared and c) after subsequent CO_2 electroreduction, and Cu foils treated with O_2 plasma at 100W for 2 minutes plus H_2 plasma at 100W for 10 minutes d) as prepared and e) after CO_2 electroreduction (1 µm scale bars). c) and e) are shown after CO_2 electroreduction at -0.9V vs. RHE for 1 hour in 0.1 M $KHCO_3$.

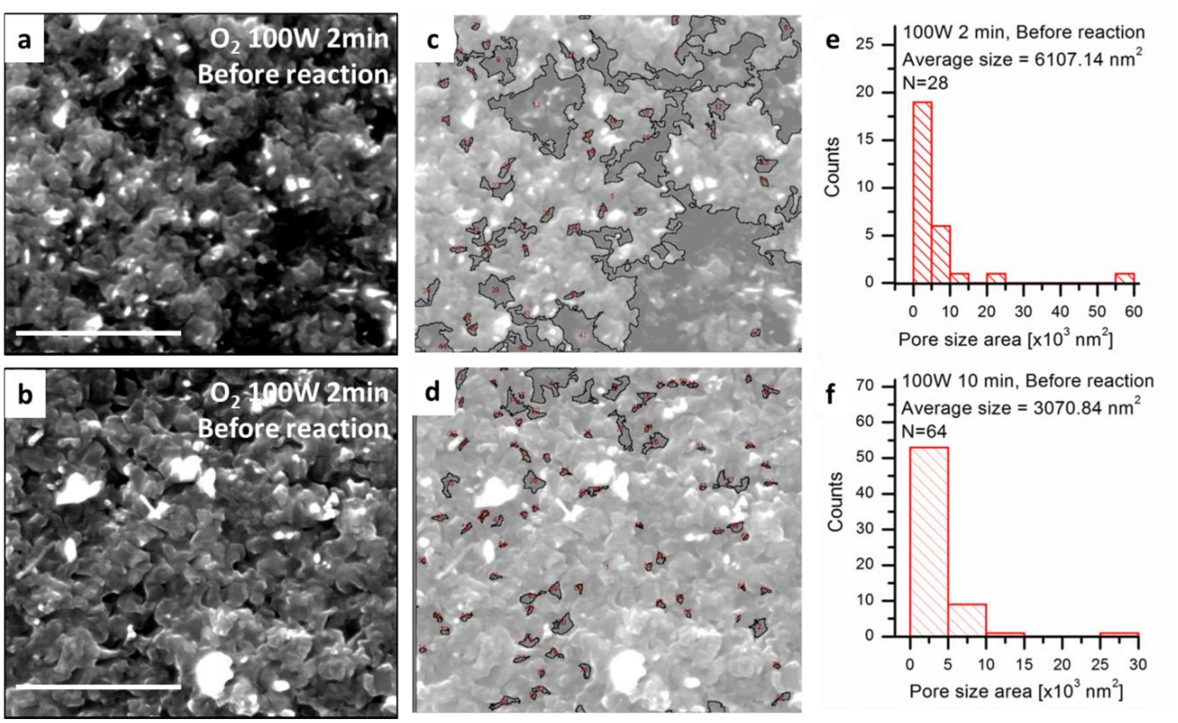

Supplementary Figure 2. Quantification of observed pores from the SEM images of the O_2 plasma treated samples at 100W for 2 min (**a**) and 100W for 10 min (**b**) before reaction (1 µm scale bars). By inspection, no visible pores were observed for the 20W for 2 min sample thus the number of pores (N) and average size were considered as 0. Image analysis was performed using ImageJ, where pores were measured manually by selecting the pores and automatically using the software. (**c**) and (**d**) show the outlined pores marked in red resulting from the automatic image analysis. Areas that do not outline a pore on the SEM were disregarded from the analysis. (**e**) and (**f**) show the resulting size distribution indicating increased porosity with plasma power and time based on the increased number of pores detected (N = 28 to 64) and decreased average pore size (6107.14 nm^2 to 3070.84 nm^2).

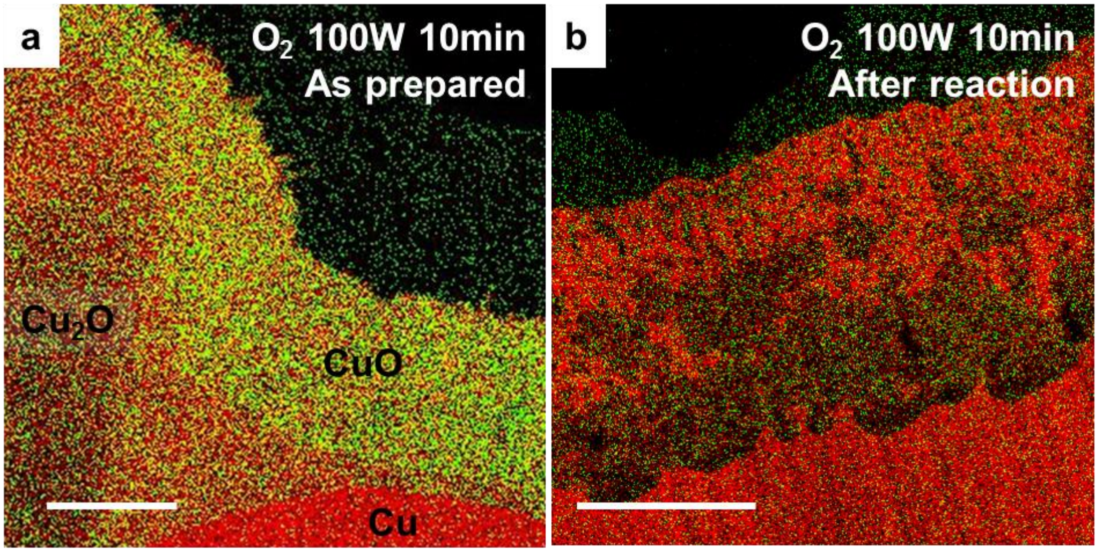

Supplementary Figure 3. EDS elemental maps of Cu foils treated with O_2 plasma for 100W 10 minutes a) before the reaction and b) after CO_2RR for 1 hour at -0.91 V vs. RHE (500 nm scale bars).

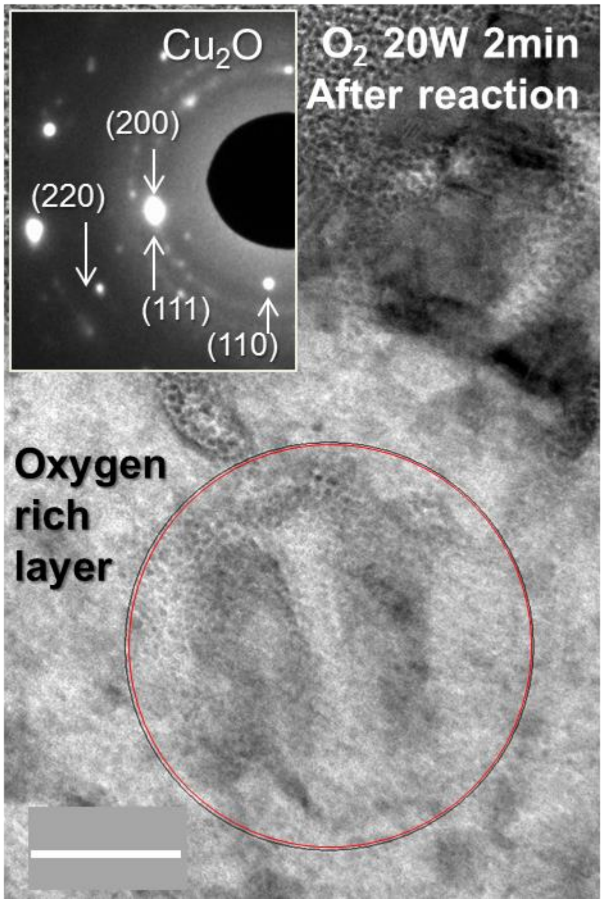

Supplementary Figure 4. HRTEM and selected area electron diffraction (SAED) analysis of the O_2 plasma 20W 2 minutes treated sample after the reaction (50 nm scale bar).

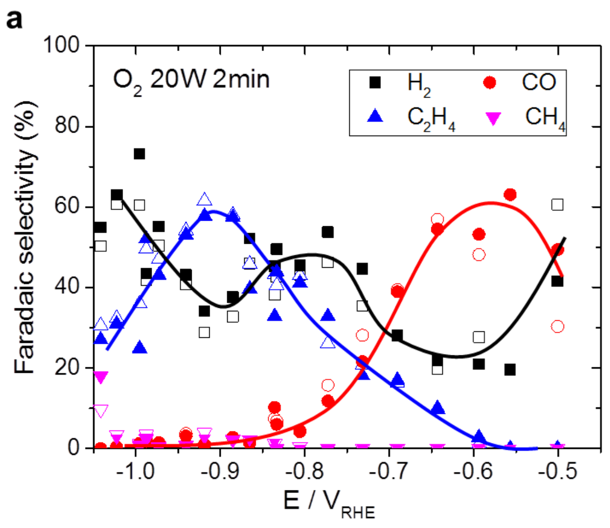

Supplementary Figure 5. Stationary Faradaic selectivity towards gas products after 10 minutes (closed symbols) and 60 minutes (open symbols) for a) O_2 20W 2 minutes and b) O_2 100W 2 minutes treated samples. Data were acquired by GC analysis of the gas phase during CO_2 electrolysis at a constant potential in CO_2 saturated 0.1 M $KHCO_3$.

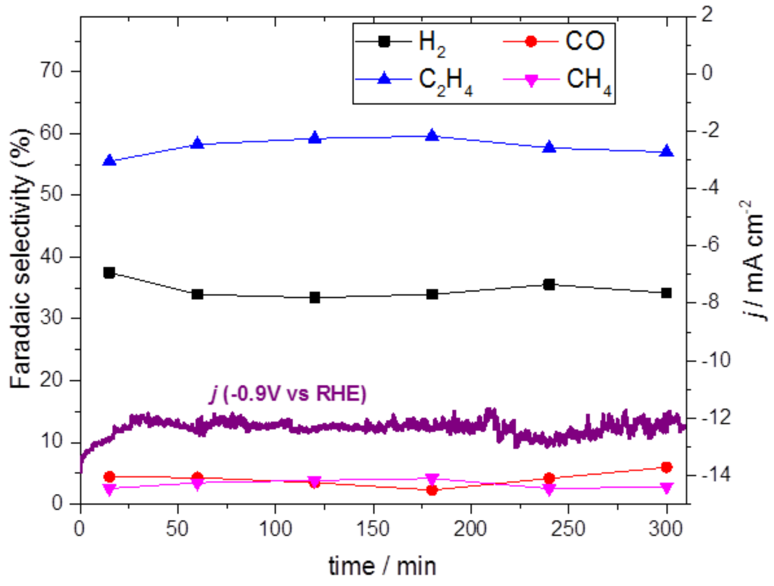

Supplementary Figure 6. Stationary current density and Faradaic selectivities of the main products of the CO_2 reduction reaction on O_2 20W 2 min plasma treated Cu foil at -0.9 V vs. RHE as a function of time.

Supplementary Figure 7. Double-layer capacitance current on the plasma treated Cu foils determined by cyclic voltammetry between 0.02 V and 0.25 V vs RHE in CO_2 saturated 0.1 M $KHCO_3$ as a function of scan rate.

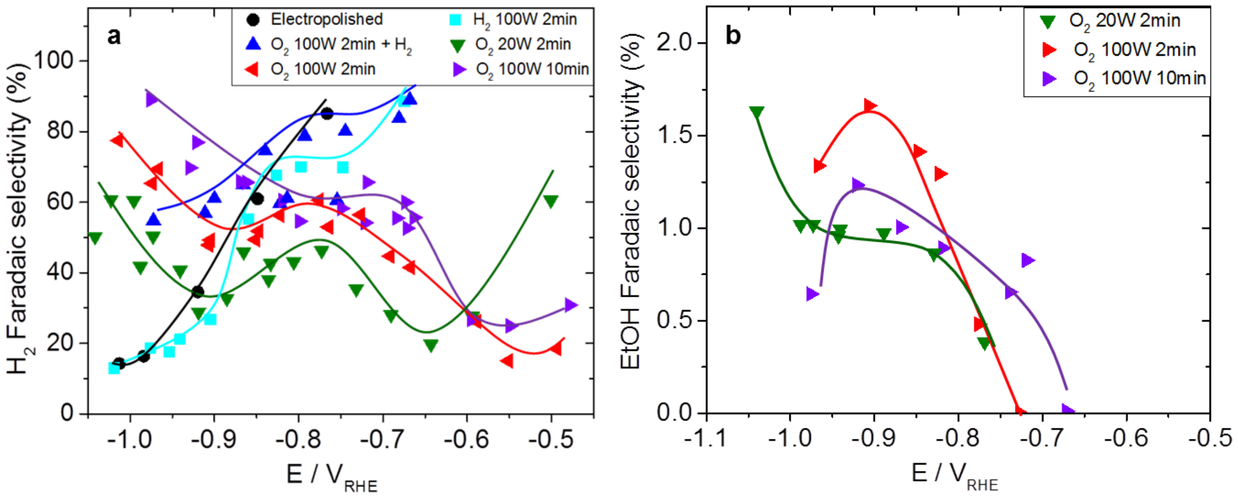

Supplementary Figure 8. Faradaic selectivity towards a) H_2 and b) ethanol as a function of electrode potentials after 60 minutes of bulk CO_2 electrolysis at a constant potential, in CO_2 saturated 0.1 M $KHCO_3$.

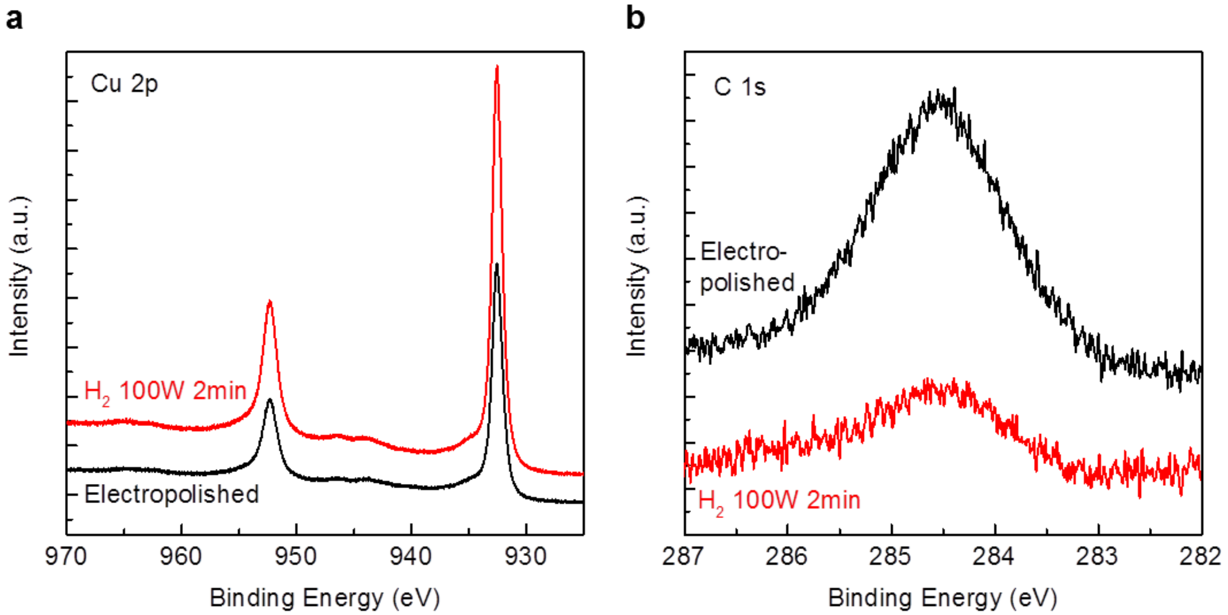

Supplementary Figure 9. XPS measurement of a) the Cu 2p and b) the C 1s regions of the electropolished Cu foil and the foil after H_2 plasma treatment at 100W for 2 minutes after *ex situ* transfer in air to our UHV XPS system.

Supplementary Table 1. Quantification of the TEM-EDS spectra acquired from the different areas of the Cu foil samples after plasma treatment/as prepared and after reactions.

Sample	Area	Cu atomic %	O atomic %	Cu:O ratio
O_2 20W 2 min As prepared	Upper layer	55.6 ± 5.1	44.4 ± 4.2	1.3:1
	Interlayer	73.0 ± 6.7	27.0 ± 2.7	2.7:1
O_2 20W 2 min After reaction	Cu rich	96.3 ± 8.9	3.4 ± 0.9	26.1:1
	Pores	80.4 ± 7.67	19.5 ± 2.6	4.1:1
O_2 100W 2 min As prepared	Upper layer	57.0 ± 5.2	43.0 ± 4.1	1.3:1
	Interlayer	73.4 ± 6.7	26.6 ± 2.7	2.8:1
O_2 100W 2 min After reaction	Cu rich	78.5 ± 7.1	21.5 ± 2.2	3.7:1
	Pores	70.5 ± 6.6	29.5 ± 3.3	2.4:1
O_2 100W 2 min + H_2 As prepared	Top surface	92.5 ± 8.5	7.5 ± 1.1	12.3:1
	Upper layer	56.3 ±5.1	43.8 ± 4.2	1.3:1
	Interlayer	72.4 ± 6.6	27.6 ± 2.8	2.6:1
O_2 100W 2 min + H_2 After reaction	Cu rich	90.7 ± 8.3	9.3 ± 1.3	9.8:1
	Pores	74.8 ± 6.8	25.2 ± 2.6	2.9:1
O_2 100W 10 min As prepared	Upper layer	57.0 ±5.2	43.0 ±4.1	1.3:1
	Interlayer	73.8 ±6.8	26.2 ±2.9	2.8:1
O_2 100W 10 min After reaction	Cu rich	90.7 ± 8.2	9.3 ± 1.2	9.7:1
	Pores	80.3 ± 7.4	19.7 ± 2.2	4.1:1

Supplementary Table 2. EXAFS fit parameters for Cu foil treated with 100W 2min O_2 plasma. Data were measured *operando* during CO_2 electroreduction at -1.2 V vs. RHE in 0.1 M $KHCO_3$. Parentheses indicate error on last digit. Before fitting, data were corrected for self-absorption.

**Sample**	**Path**	**N**	**r (Å)**	**σ^2 (Å^2)**
As prepared	Cu-Cu (Cu^0)	1.4 (3)	2.57 (1)	0.004 (2)
	Cu-O (Cu_2O)	1.2 (2)	1.89 (1)	0.004 (2)
	Cu-Cu (Cu_2O)	11 (3)	3.11 (2)	0.029 (4)
15 minutes reaction	Cu-Cu (Cu^0)	6 (2)	2.54 (2)	0.004 (2)
	Cu-O (Cu_2O)	0.9 (7)	1.87 (6)	0.000 (9)
1 hour reaction	Cu-Cu (Cu^0)	12 (set)	2.53 (1)	0.0066 (7)

Supplementary Table 3. Double-layer capacitance and roughness factors for Cu foils after plasma treatments, following the method from Li et al.[1] Double layer capacitance was estimated as the slope obtained in Supplementary Figure 5. The surface roughness factor for electropolished Cu was defined to be 1, and it was used to determine the roughness for the other foils as follows:

$$Roughness\ factor = \frac{Capacitance}{0.0474} \tag{1}$$

Sample	Capacitance mF	Roughness Factor
Electropolished	0.0474	1.0
H_2 100W 2min	0.0705	1.5
O_2 20 W 2min	1.25	26.4
O_2 100 W 2min	2.07	43.7
O_2 100 W 2min + H_2	2.29	48.3
O_2 100 W 10min	4.25	89.7

Supplementary References

1 Li, C. W. & Kanan, M. W. CO_2 reduction at low overpotential on Cu electrodes resulting from the reduction of thick Cu_2O films. *J. Am. Chem. Soc.* **134**, 7231-7234 (2012).
