# Supplementary material for: Highly selective plasma-activated copper catalysts for carbon dioxide reduction to ethylene
Source: Nat Commun. 2016 Jun 30;7:12123. doi: 10.1038/ncomms12123 (PMC4931497; doi:10.1038/ncomms12123)
Supplement: Peer Review File [file ncomms12123-s2.pdf]

Editorial Note: this manuscript has been previously reviewed at another journal that is not operating a transparent peer review scheme. This document only contains reviewer comments and rebuttal letters for versions considered at Nature Communications.

REVIEWERS' COMMENTS:

Reviewer #1 (Remarks to the Author):

Comment on the manuscript NCOMMS-16-02800A

Authors' work has focused on electrochemical reduction of CO2 to selectively produce C2H4 using the Cu-based catalysts improved with plasma. The results demonstrate that the roughness of oxide-derived Cu catalysts plays only a partial role in determining the catalytic performance, while the presence of Cu(I) is a key for lowering the onset potential and enhancing ethylene selectivity. I think this work has a considerable originality and interest. The approach has validity, and the quality of data is high. The conclusions are valid. References are well arranged. This manuscript has been largely improved and no improvement will be suggested.

Reviewer #2 (Remarks to the Author):

I have read and evaluated the response submitted by the authors to concerns raised by reviewers. I believe the authors addressed all issues raised either in their response, revision of the manuscript, and additional information in supporting information. Therefore, I enthusiastically recommend publication of this manuscript.

Reviewer #3 (Remarks to the Author):

This paper is now ready for publication
